# Temperature- and Time-Resolved Gas Release Coupled with Degradation of an Overheated Medium-Voltage Cable PVC Outer Jacket

**DOI:** 10.3390/polym18141749

**Published:** 2026-07-17

**Authors:** Xiaobo Chen, Wenchang Zhang, Peng Ru, Jia Zhang

**Affiliations:** 1 Jiuquan Power Supply Company, State Grid Gansu Electric Power Company, Jiuquan 735099, China; 15693760608@163.com (X.C.); 15293481050@163.com (W.Z.); rupeng18706919715@163.com (P.R.); 2 School of Space Science and Technology, Xidian University, Xi’an 710126, China

**Keywords:** PVC cable outer jacket, overheating, decomposition gases, plasticizer migration, GC-MS, dielectric deterioration

## Abstract

Overheating of polymeric cable materials is a major contributor to insulation aging, electrical failure, and fire risk in power systems, particularly in densely installed urban underground cable corridors where heat dissipation is limited and early-stage defects are difficult to identify. Although gas-detection approaches are promising for non-invasive overheating monitoring, their practical value depends on identifying material- and lay-er-specific volatile products and clarifying how their release evolves with temperature and time. Herein, volatile products released from the PVC outer jacket of a YJV22-8.7/15 kV-3 × 185 medium-voltage cable were investigated using headspace gas chromatography–mass spectrometry (GC-MS). Temperature- and time-dependent evolution was estimated for selected marker species, and the associated degradation behavior was correlated with chemical/structural and electrical changes using ATR-FTIR, KPFM, and dielectric measurements. The number and observed headspace levels of organic components increased substantially with severe overheating, reaching more than 20 dominant components at 200 °C. 2-Ethylhexanol (2-EH) was observed across the studied range and reached approximately 300 × 10^−6^ (volume fraction) at 200 °C for 60 min while remaining at or below approximately 50 × 10^−6^ at temperatures up to 140 °C. Benzene was observed mainly at severe overheating, whereas DOTP was first observed at 140 °C among the tested conditions, reaching approximately 40 × 10^−6^ at 140 °C for 5 min and exceeding 300 × 10^−6^ under more severe conditions. KPFM showed surface roughness increasing from 7.70 to 43.39 nm, and the real permittivity increased by up to 13.9% at 50 Hz. These results provide a temperature- and time-resolved headspace dataset for the tested cable outer jacket and relate its organic-gas profile to surface and dielectric changes.

## 1. Introduction

Cables are fundamental equipment for transmitting and distributing electrical energy in power systems and are deeply integrated into modern industrial production and daily life. The rapid increase in electricity demand has led to heavier cable loads and higher operating temperatures, introducing numerous safety hazards. At the same time, urban cable burial projects and city landscape requirements have objectively worsened cable heat-dissipation conditions, making cable overheating faults increasingly prominent [1]. Factors including aging during service, operation at currents beyond the steady-state rating, conductor damage inside the cable, and an insufficient conductor cross-section can all cause an overload during operation [2,3]. In urban power grids, large numbers of cables are frequently bundled and laid in underground tunnels or cable shafts within buildings. The confined spaces and mutual shielding among cables make latent overheating faults difficult to detect, causing cables to operate at elevated temperatures, accelerating insulation aging, and potentially leading to breakdown, short circuits, or fires. Once ignited, bundled cables can burn and propagate rapidly across the route, with spread rates of up to 20 m/min, causing severe consequences [4].

Overheating is one of the main causes of cable ignition and combustion [5]. Bundled installation and the flammability of polymeric cable materials mean that, once an accident occurs, it can become a chain fire affecting a wide area, causing major social impact and economic loss. Therefore, diagnosing cable overheating faults is important for preventing electrical fires and ensuring the safe operation of power systems.

Gas detection judges whether an electrical fire or precursor overheating event has occurred by detecting gases produced by cable thermal decomposition. Because decomposition gases are generated earlier than smoke particles, this method theoretically has strong potential for early detection of cable-overheating [6]. Gases have good mobility, which allows the sensing device to be separated from the overheating location, reduces the influence of obstacles and shielding, provides strong immunity to electromagnetic interference, and enables non-contact detection [7]. However, response time and warning capability depend heavily on the selected target gases [8]. Conventional fire-detection targets, such as CO, CO_2_, HCN, and HCl, cannot by themselves provide reliable cable-overheating information because the characteristic gases of early overheating remain unclear [9]. It is therefore necessary to identify the decomposition products of cable overheating, clarify their patterns of variation, and establish correlations between gas release and severity of overheating.

Poly(vinyl chloride) (PVC) is widely used as an insulating and jacket material for cables. When heated, PVC cable insulation releases various gases that are closely related to temperature. O’Mara and colleagues used pyrolysis–gas chromatography–mass spectrometry (Py-GC-MS) to detect PVC decomposition products at 600 °C in an inert atmosphere; they reported a series of aliphatic and aromatic hydrocarbons, with benzene as the main organic compound [10]. Ohrbach et al. used thermogravimetry–mass spectrometry (TG-MS) to study PVC pyrolysis products in static air and detected abundant HCl, infer-ring that chain-scission fragments and aromatic compounds were also present [11]. Knumann used TG-MS to determine the pyrolysis gas products of waste plastics and PVC products under nitrogen; when the temperature exceeded 250 °C, gases such as HCl, benzene, and unsaturated hydrocarbons gradually appeared [12]. Wang et al. studied cable pyrolysis behavior using TG-FTIR and found that pyrolysis gases were mainly generated in the 500–1000 K range, including CO_2_, HCl, and gases containing C-H, CH_2_, and C-Cl functional groups [13]. These studies revealed the composition of mixed gases after high-temperature PVC pyrolysis, but limited attention was paid to the temperature dependence of decomposition gases in the lower overheating range.

Most related studies focus on gas components produced by high-temperature pyrolysis of PVC materials above 200 °C, often using PVC polymers, plastic products, or discarded circuit boards as model objects [14,15,16,17]. Relatively few studies address decomposition gases from actual cable-grade PVC insulation under overheating conditions below 200 °C. Because the formulation of cable PVC insulation differs from that of consumer PVC plastics, pyrolysis gases from generic PVC products cannot be directly extrapolated to cable-overheating scenarios. Benes’ results indicate that decomposition gases from PVC cable insulation in the 200–340 °C range include not only PVC pyrolysis products such as HCl, CO_2_, and benzene, but also organic compounds derived from plasticizers added to cable insulation to satisfy power-system application requirements [18].

Overall, previous TG-MS, TG-FTIR, and Py-GC/MS studies have provided important information on PVC pyrolysis products and thermal-degradation pathways, especially under high-temperature or thermal-analysis conditions. However, those studies were mainly designed to reveal general polymer degradation chemistry, and they provide limited information for early cable-overheating diagnosis. In practical cable corridors, the key question is not only what gases may appear during complete pyrolysis, but which volatile markers can be detected during the lower-temperature overheating stage, how their concentrations evolve with heating temperature and duration, and whether these gases can be linked to insulation structural and dielectric deterioration [19]. Therefore, a formulation-specific study using actual cable-grade PVC insulation is still needed to bridge polymer degradation chemistry and gas-based overheating warning.

Recent studies have examined gas-assisted diagnosis of overheated cable materials using related but non-identical systems. Zhang et al. analysed actual inner and outer PVC cable layers using programmed TG-FTIR, GC/GC-MS, electrical-stress experiments, and molecular simulations [3]. Sugitani et al. studied processing-related volatile markers from a commercial 600 V PVC cable and combined GC-MS peak-area analysis with SnO2 sensing and classification [9]. Liu et al. evaluated different PVC and XLPE cables using GC-MS screening and odor-sensor arrays [20], Meng et al. classified thermal states of a ZC-BV10 PVC sheath from gas-sensor-array responses [21], and Cao et al. developed staged warning states for a polyethylene high-voltage-cable outer sheath [22].

Table 1 compares the cable construction and analyzed layer, temperature–time protocol, analytical output, reported gas markers, and threshold or validation status. Sugitani et al. combined PVC insulation and sheath fragments from a 600 V cable, whereas the present specimens were taken only from the PVC outer jacket of a YJV22-8.7/15 kV-3 × 185 medium-voltage armoured cable. Their dominant reported markers were acetophenone, 2-phenyl-2-propanol, and 2-EH, whereas the present profile comprised 2-EH, DOTP, DEHA, 2-EBe, 2-EBu, and benzene. Thus, 2-EH was common to both studies, while the accompanying compounds and their relative prominence differed with the cable construction, analysed layer, additive composition, and sampling protocol.

Against this background, the present study examines volatile products released from the PVC outer jacket of a YJV22-8.7/15 kV-3 × 185 medium-voltage cable over 90–200 °C. Headspace GC-MS response conversion is used to estimate temperature- and time-dependent levels of selected organic markers, and the gas trends are evaluated together with ATR-FTIR, KPFM, and dielectric measurements obtained from the same cable material. The analysis therefore describes the temperature–time–gas–surface–property relationships observed for this specific cable layer under the stated laboratory conditions.

## 2. Materials and Methods

### 2.1. Cable Insulation Samples

Cable-grade PVC insulation material was used as the research object. PVC insulation materials are widely used for the outer jackets of high- and low-voltage cables because they offer flexibility, durability, impact resistance, and processability, thereby satisfying both mechanical and electrical performance requirements. PVC insulation materials are mainly composed of PVC polymer. They are formulated with plasticizers, stabilizers, lubricants, fillers, and other additives in different proportions [23,24]. PVC insulation materials exhibit excellent electrical and insulating properties, low flammability, toughness and durability, and good abrasion resistance, making them widely used raw materials in cable manufacturing.

**Figure 1:**
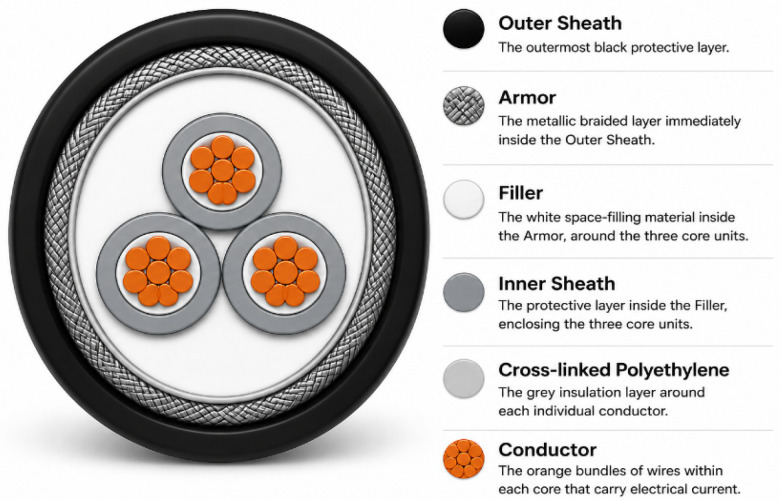
Structure of the investigated cable and sampling position of the PVC outer-jacket insulation.

The cable structure is shown in Figure 1. The PVC specimens used in this study were taken from the outer jacket of a YJV22-8.7/15 kV-3 × 185 cable(Jiangsu Shangshang Cable Group, Changzhou, China). The manufacturer did not disclose the exact commercial compound formulation. Table 2 summarizes typical component ranges for cable-grade PVC.

The PVC polymer chains have relatively short intermolecular distances and strong intermolecular interactions, rendering PVC a solid and brittle material. Moreover, PVC generally has poor thermal stability and limited resistance to light/UV exposure; thus, it can degrade at relatively low temperatures and cannot be used directly for cable production [25].

Plasticizers are the most commonly used and largest fraction of additives in PVC, enabling the PVC polymer to become softer and more flexible [26,27]. At present, approximately 50 plasticizers are used in commercial applications. High-molecular-weight phthalate and terephthalate-type plasticizers are mainly used for cable PVC insulation during manufacturing, including phthalate plasticizers (DOP and DINP) and non-phthalate plasticizers (e.g., DOTP). Due to health concerns, phthalate-type plasticizers have been progressively restricted or banned by the European Union and related regulatory authorities. They are replaced by di(2-ethylhexyl) terephthalate (DOTP), which provides comparable plasticization performance while presenting lower risks to health and the environment.

Stabilizers are used to prevent chain-decomposition reactions during the manufacturing and processing of PVC. Owing to cost considerations, their content is typically low. Various stabilizers are commonly used in cable-grade PVC insulation, including metal soaps, metal salts, and organometallic compounds [28].

Fillers mainly reduce cable manufacturing costs, improve impact resistance, and enhance electrical and physical properties [29]. Calcium carbonate is the most widely used filler [30].

### 2.2. Gas Component Analysis Using GC-MS

When a cable overheats, the insulation material undergoes PVC thermal decomposition and plasticizer migration. The chemical reactions and physical transport processes involved the release of corresponding gas components. These gases are important for clarifying the gas-generation pathway of cable overheating and related physicochemical processes.

Gas chromatography-mass spectrometry (GC-MS) combines the separation capability of gas chromatography with the identification capability of mass spectrometry. It can separate target compounds from complex mixtures and rapidly identify them. GC-MS consists of a gas chromatograph (GC), which separates components in a mixture, and a mass spectrometer (MS), which identifies each separated component.

**Figure 2:**
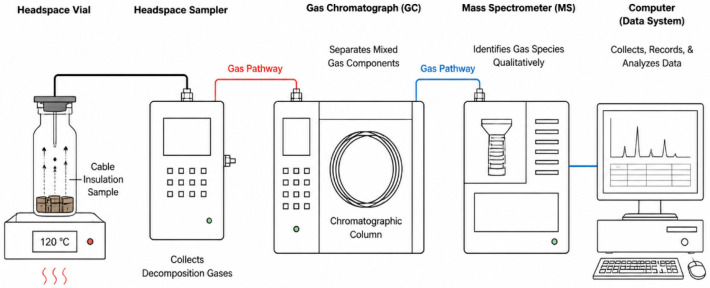
Schematic diagram of the cable-overheating decomposition-gas detection system used for GC-MS qualitative and quantitative analysis.

A schematic diagram of the cable-overheating decomposition-gas detection system is shown in Figure 2. In the system, a heated headspace vial containing the cable insulation sample is connected to a headspace sampler that collects decomposition gases. The gases then enter the GC through the gas pathway, are separated on the chromatographic column, and are transferred to the MS for qualitative identification. The data are recorded and analyzed using a computer system.

An Agilent GC-MS system consisting of a 7890B gas chromatograph (Agilent Technologies, Santa Clara, CA, USA) and a 5977B mass spectrometric detector (Agilent Technologies, Santa Clara, CA, USA) was used to identify organic products released via cable material under overheating conditions. An HP-5MS capillary column (30 m × 0.25 mm × 0.25 um) (Agilent Technologies, Santa Clara, CA, USA) was selected for the qualitative analysis and external-standard response conversion of volatile and semi-volatile organic compounds. The column uses 5% phenyl and 95% methylpolysiloxane during the stationary phase and provides suitable separation of the alcohol, ester, and aromatic markers considered in this study.

The GC oven program was as follows: the initial temperature was set to 40 °C and held for 5 min; increased to 60 °C at 5 °C/min and held for 1 min; increased to 90 °C at 2 °C/min and held for 1 min; and finally, increased to 280 °C at 10 °C/min and held until the end of the analysis.

Full-scan acquisition mode was first used to detect a wide range of mass fragments and preliminarily analyze mixture composition. Because the peaks of overheating decomposition gases in the total ion chromatogram (TIC) were much smaller than those of background air, the scan range was set to *m*/*z* 35–500 to avoid background interference. The gas species present in the TIC were identified using the mass-spectral database released by the National Institute of Standards and Technology (NIST). High-purity helium was used as the carrier gas at a constant flow rate of 1.0 mL/min. For the six target compounds used in quantitative analysis, identification was based on both NIST library matching and comparison with external-standard retention behavior.

A single-point external-standard response conversion was used to estimate headspace levels of selected overheating products. The chromatographic response of an unknown sample was converted using the response measured for a standard of known concentration, as expressed in Equations (1) and (2):

C_i_ = f(A_i_) (1)

C_i_ = C_s_ × A_i_/A_s_ (2)

where C_i_ is the concentration of target component i; A_i_ is the chromatographic peak amplitude or peak height of target component i; C_s_ is the calibration concentration of the standard component; and A_s_ is the chromatographic peak amplitude or peak height of the standard component.

After the chromatographic peak of each target compound was identified, its peak height was converted using the corresponding external-standard response factor. The resulting values are reported as estimated laboratory headspace volume fractions under the specified sampling and GC-MS conditions.

The six principal target compounds were evaluated using external standards at the following levels: DOTP (528 × 10^−6^), 2-EH (594 × 10^−6^), benzene (50 × 10^−6^), DEHA (630 × 10^−6^), 2-EBu (205 × 10^−6^), and 2-EBe (70 × 10^−6^). Each standard and each overheating condition was measured ten times; the mean peak height was used for response conversion, and the error bars represent the standard deviation of the ten measurements.

### 2.3. Thermal, Surface, Spectroscopic, and Electrical Characterization

The PVC insulation samples were taken from the cable’s outer jacket (YJV22-8.7/15 kV-3 × 185). The sample dimensions were approximately 3 × 3 × 0.5 cm. Samples were placed in glass Petri dishes inside a DHG-9055A oven. The initial set temperature was 50 °C; it then increased in 20 °C steps until the insulation material completely melted. Each temperature was maintained for approximately 5 min, and the physical changes were observed through the oven window and recorded. Dry synthetic air (O_2_ + N_2_) was used as the test atmosphere to approximate the oxygen-containing environment during cable overheating, with a gas flow rate of 40 mL/min. Aluminum oxide (Al_2_O_3_) crucibles were used.

During thermal degradation of PVC insulation, surface microstructure and electrical characteristics evolve together. Kelvin probe force microscopy (KPFM), an extension of atomic force microscopy, was used to characterize the surface after thermal degradation. KPFM enables simultaneous nanoscale measurement of surface morphology and surface potential based on electrostatic-force interactions between the probe and the sample. Three-dimensional surface morphology was recorded in tapping mode.

KPFM testing was performed using a Shimadzu SPM-9700HT AFM system (Shimadzu Corporation, Kyoto, Japan). Sheet-like PVC samples were fixed onto a metallic stage using silver conductive adhesive tape. High-conductivity probes were selected, and tapping mode was used at a scan rate of 1 Hz over a 1 µm × 1 um area with a resolution of 512 × 512 pixels.

Infrared spectroscopy was used for material characterization and identification. An ATR-FTIR system was used to examine changes in surface functional groups and chemical substances of PVC insulation after overheating. Samples were cut from the outer jacket of the same YJV22-8.7/15 kV-3 × 185 cable, sized at approximately 0.5 × 4 × 0.3 cm. Blank control specimens and thermally oxidized specimens aged for 84 h at 120 °C were prepared. The ATR-FTIR system was a Bruker TENSOR II with a ZnSe ATR crystal (Bruker Optics GmbH & Co. KG, Ettlingen, Germany).

Before testing, the ATR crystal was cleaned with isopropanol and dried. An air spectrum was collected as the background spectrum, and the sample was then brought into tight contact with the crystal using the ATR clamp. Spectra were collected over 500–4000 cm^−1^ with a resolution of 0.2 cm^−1^ and a scan rate of 32 scans/min. In infrared spectroscopy, the relation between absorbance A at a specific wavelength and the concentration c of the target analyte is described by the Beer–Lambert law [31,32]:

A = log_10_(I_0_/I) = εlc (3)

where I_0_ is the incident light intensity, I is the transmitted or reflected light intensity used in the ATR configuration, A is absorbance, ε is the molar absorption coefficient, l is optical path length, and c is the concentration of the specific analyte.

## 3. Results

### 3.1. Identification of Decomposition Gases

Using the GC-MS system, gas components produced during the overheating of cable PVC insulation at 90–200 °C were detected. During overheating, the majority of generated gas components were composed of C, H, and O. Based on elemental composition, they can be broadly classified as hydrocarbons and hydrocarbon–oxygen compounds, consistent with the chemical composition of PVC insulation. According to molecular structure, they can be divided further into organic compounds containing benzene rings and those without ring structures.

Among the organic products observed during cable overheating, some were consistent with additive-related compounds, whereas others were consistent with products of PVC-chain transformation. DOTP and DEHA are plasticizers used in PVC formulations, and 2-EH is a feedstock and possible transformation product associated with 2-ethylhexyl ester plasticizers. Benzene and other aromatic compounds can form through established PVC dehydrochlorination, polyene evolution, scission, crosslinking, and cyclization pathways [33,34,35].

**Figure 3:**
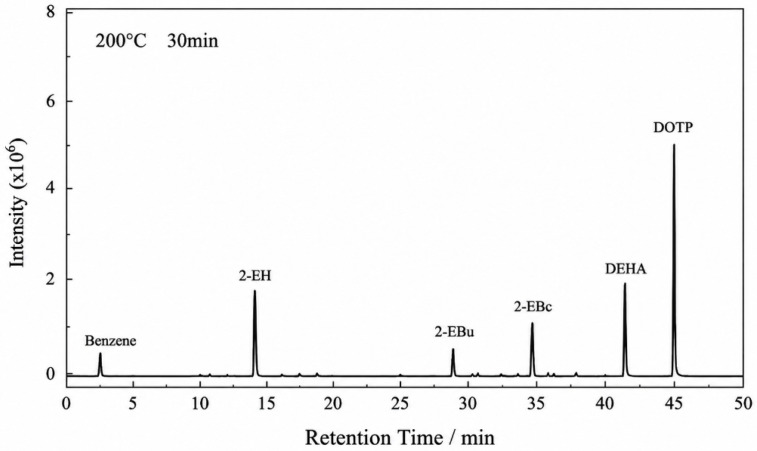
Chromatograms of decomposition gases released from cable PVC insulation after overheating at 200 °C for 30 min.

Figure 3 shows chromatograms of decomposition-gas components produced by overheating cable PVC insulation at 200 °C for 30 min. Based on the relative peak amplitudes, the main decomposition-gas components generated during cable overheating are 2-EH, DOTP, DEHA, 2-EBe (2-ethylhexyl benzoate), 2-EBu (2-ethylhexyl butyrate), and benzene.

No separate HCl peak was reported in the present GC-MS chromatograms. This should not be interpreted as evidence that PVC dehydrochlorination was absent. HCl is a highly polar, inorganic, and reactive gas; by contrast, the selected HP-5MS/headspace GC-MS method was optimized for volatile and semi-volatile organic markers including 2-EH, DOTP, DEHA, 2-EBe, 2-EBu, and benzene. In addition, the tested 90–200 °C range, the dry-air headspace pathway, and formulation additives/stabilizers may influence recoverable HCl in the organic GC-MS channel. Therefore, HCl is discussed here as a mechanistic product of PVC dehydrochlorination, while the quantitative diagnostic analysis focuses on organic marker gases that are more compatible with the selected analytical method and field VOC sensing.

Based on the GC-MS and ATR-FTIR observations, the organic products in Figure 3 were interpreted as two operational groups. The first group, represented by 2-EH, DOTP, and DEHA, is consistent with additive migration, volatilization, and possible additive transformation. The second group, represented by benzene, is consistent with the chemical transformation of the PVC matrix at higher severe overheating severity. This operational grouping summarizes the marker evolution observed under the tested conditions.

**Figure 4:**
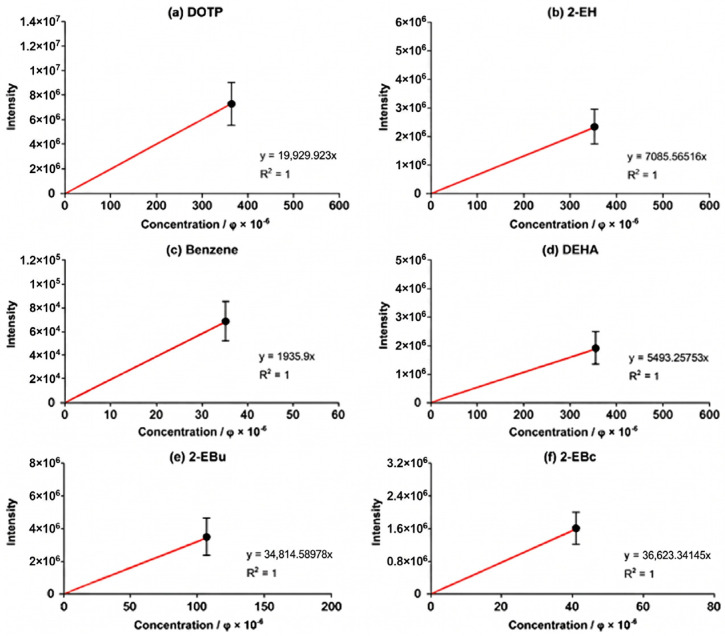
External-standard response-factor representation for the six organic marker compounds considered in the headspace analysis.

The six principal organic products considered in the quantitative plots, namely DOTP, 2-EH, benzene, DEHA, 2-EBu, and 2-EBe, were evaluated using the external-standard response conversion described in Section 2.2. Figure 4 shows the corresponding response-factor representation. Each standard level was measured ten times, and the error bars represent the standard deviation.

### 3.2. Temperature-Dependent Variation in Gas Species

**Figure 5:**
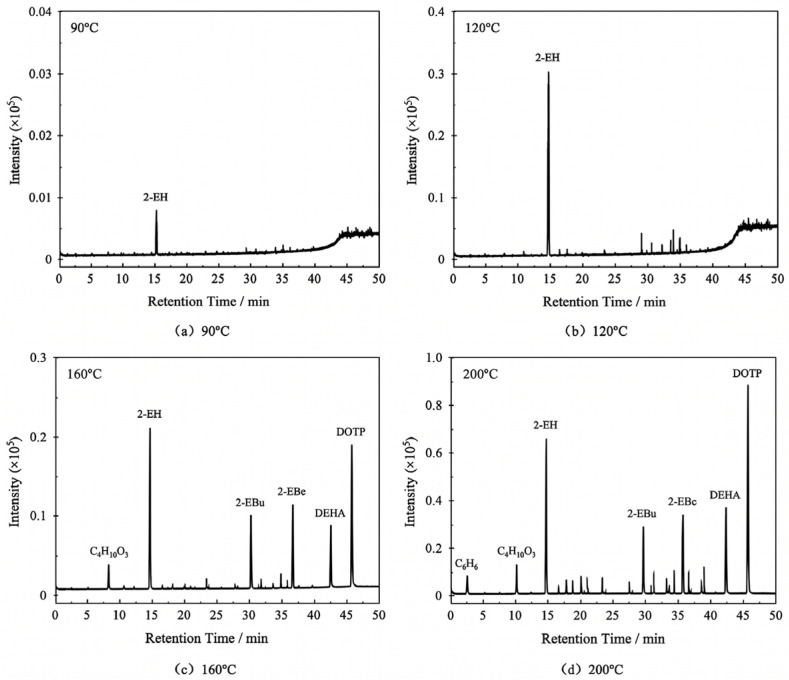
Chromatograms of decomposition gases from cable PVC insulation at different overheating temperatures.

Figure 5 shows the total ion chromatograms of decomposition gases released from cable PVC insulation after exposure to different overheating temperatures for 5 min. Each peak represents one gas component.

The number and estimated headspace levels of the observed organic products increased with temperature. At 90 °C, only trace components were observed. At 120 °C, additional components appeared; above 120 °C, both the number of observed peaks and their estimated levels increased more rapidly. At 200 °C, both reached their maximum within the tested 90–200 °C range. These observations show condition-dependent differences in the volatile profile.

Therefore, the observed organic profile of the tested outer jacket was closely related to the overheating temperature under the present experimental configuration. Lower-temperature conditions produced fewer and lower-level peaks, whereas higher-temperature conditions produced more components and higher estimated headspace levels, with more than 20 dominant components observed at 200 °C.

### 3.3. Concentration Evolution of Marker Gases

Because decomposition gases are diverse and their concentrations differ significantly, it is not practical to analyze every detected component in detail. Some gases remain at very low concentrations even at 200 °C, while others correspond to compounds without clear functional roles in PVC insulation. Based on the six main overheating gases identified above, this section examines the behavior of DOTP, 2-EH, DEHA, 2-EBe, 2-EBu, and benzene in the 90–200 °C range.

**Figure 6:**
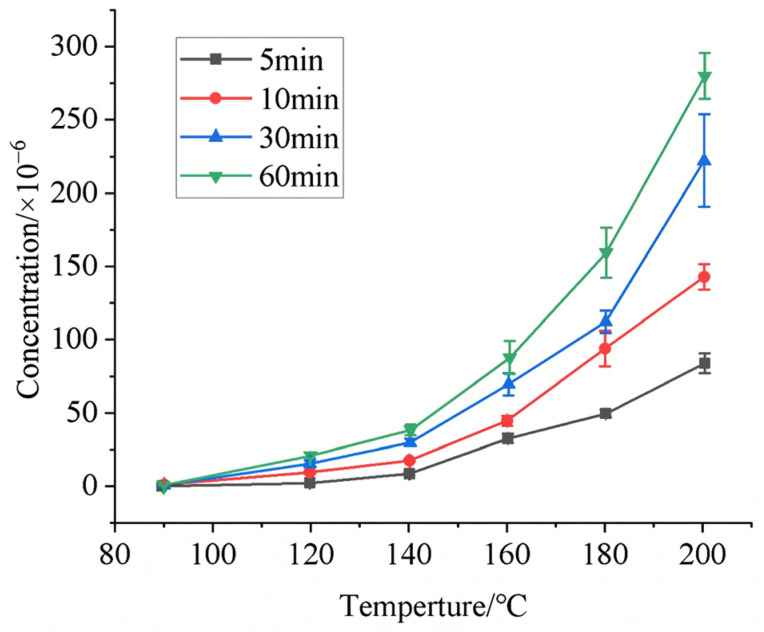
Relationship between 2-EH concentration and overheating temperature/duration.

The relationship between the estimated 2-EH headspace level, overheating temperature, and duration is shown in Figure 6. 2-EH was observed under all tested 90–200 °C conditions. For a given duration, its estimated level increased with temperature, and longer exposure produced a stronger increase. The maximum estimated level was approximately 300 × 10^−6^ (volume fraction) at 200 °C for 60 min. At temperatures up to 140 °C, the estimated level increased slowly and remained below approximately 50 × 10^−6^, with only a very small response at 90 °C.

Thus, 2-EH is a useful candidate marker for the tested cable outer jacket because it was observed throughout the 90–200 °C range and showed a strong positive association with temperature and duration.

**Figure 7:**
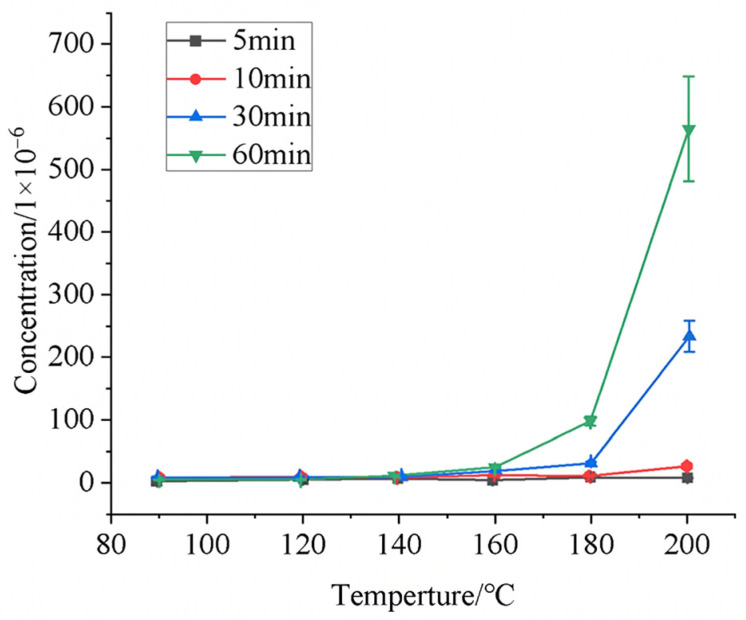
Relationship between benzene concentration and overheating temperature/duration.

The relationship between the estimated benzene headspace level, overheating temperature, and duration is shown in Figure 7. Benzene was first observed at approximately 140 °C among the tested conditions and was nearly absent below 120 °C. Where observed, its estimated level increased with temperature and rose more rapidly after longer exposure. This delayed appearance is consistent with the progression of the established PVC dehydrochlorination–polyene–aromatization sequence [33,34,35]. The 140 °C value is reported as the observed laboratory onset level within the tested temperature grid.

**Figure 8:**
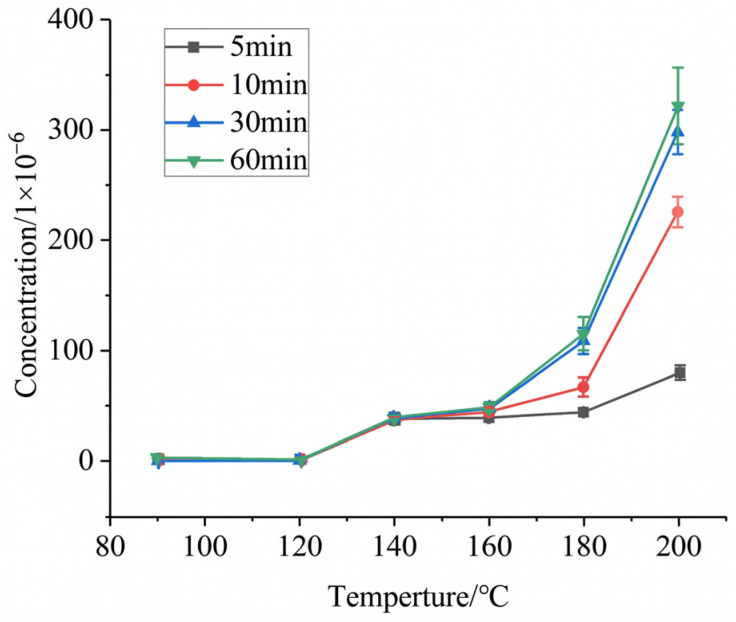
Relationship between DOTP concentration and overheating temperature/duration.

The relationship between the estimated DOTP headspace level, overheating temperature, and duration is shown in Figure 8. DOTP was first observed at 140 °C among the tested conditions. For a given duration, its estimated level generally increased with temperature, although the trend varied with duration and the 200 °C results may also reflect decomposition or re-condensation [36,37,38]. DOTP reached approximately 40 × 10^−6^ after 5 min at 140 °C and exceeded 300 × 10^−6^ under more severe conditions. These observations identify DOTP as a material-specific higher-severity marker candidate.

**Figure 9:**
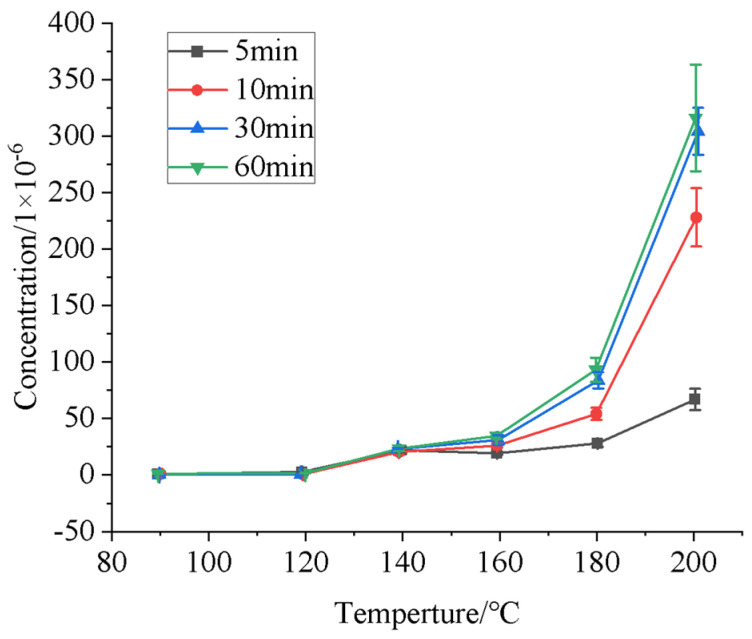
Relationship between DEHA concentration and overheating temperature/duration.

The relationship between the estimated DEHA headspace level, overheating temperature, and duration is shown in Figure 9. DEHA exhibited a trend similar to DOTP but at a lower estimated level. Because the exact manufacturer formulation was not disclosed, the observation of DEHA is treated as analytical evidence of a DEHA-related component in the tested jacket rather than proof of its specified formulation fraction.

Therefore, DEHA may serve as a supplementary marker candidate for this tested cable material at higher overheating severity.

**Figure 10:**
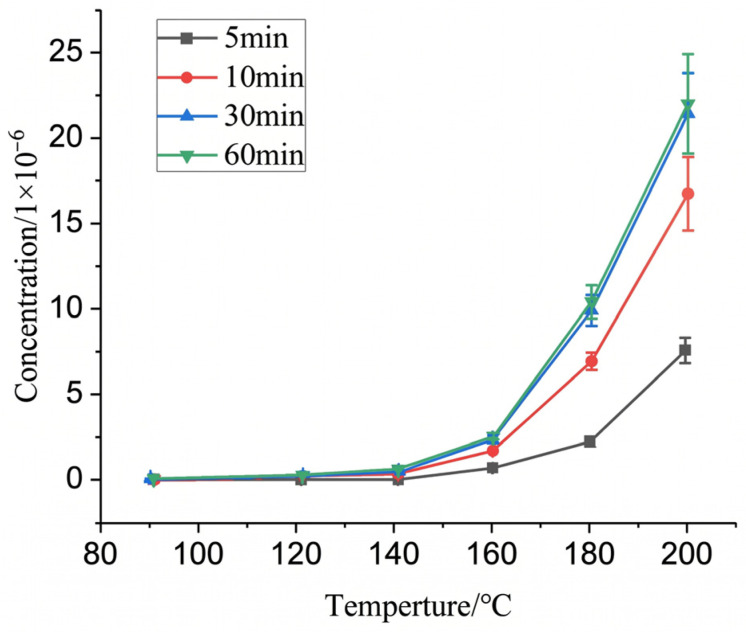
Relationship between 2-EBe concentration and overheating temperature/duration.

**Figure 11:**
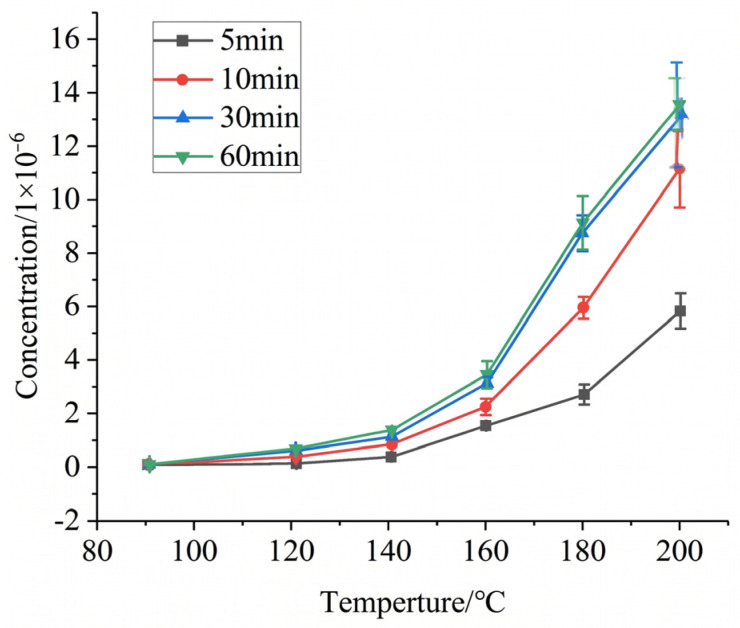
Relationship between 2-EBu concentration and overheating temperature/duration.

The relationships between the estimated 2-EBe and 2-EBu headspace levels, overheating temperature, and duration are shown in Figure 10 and Figure 11. Both components were observed within the tested range and increased most clearly above 140 °C. Their maximum estimated levels did not exceed approximately 20 × 10^−6^ and 15 × 10^−6^, respectively, and were substantially lower than those of 2-EH and DOTP.

Accordingly, 2-EBe and 2-EBu are treated as secondary components in the present material-specific profile rather than primary warning markers.

## 4. Experimentally Supported Coupled Degradation Pathway

### 4.1. Chemical and Microstructural Degradation

**Figure 12:**
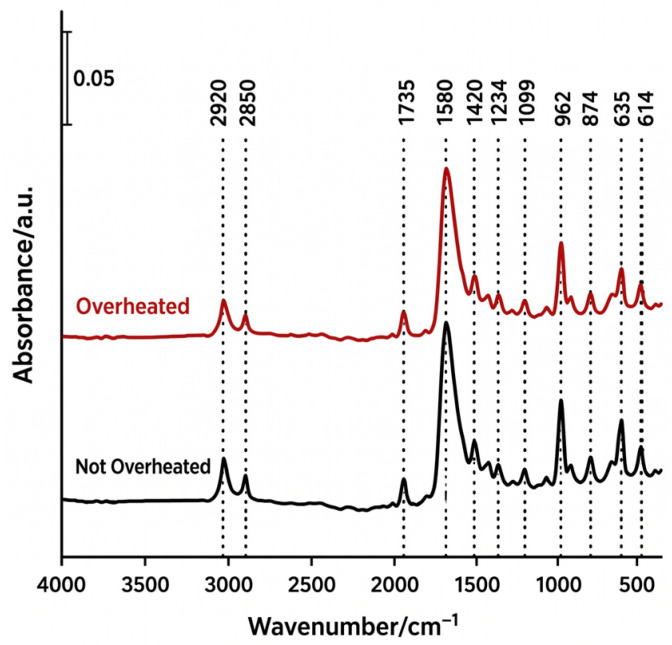
Infrared spectral changes in cable insulation before and after overheating at 120 °C.

Figure 12 compares the infrared spectra of cable PVC insulation before and after exposure to overheating. The IR absorption peaks and corresponding groups indicate that the cable insulation is a blend composed of PVC polymer and multiple additives. Three absorption peaks in the 600–700 cm^−1^ region are assigned to stretching vibrations of C-Cl single bonds. The absorption peak at 1420 cm^−1^ represents the -CH_2_-CHCl- structure, the peaks at 1254 and 1334 cm^−1^ correspond to CH bending vibrations of -CHCl-, the peak at 1099 cm^−1^ corresponds to stretching of the main-chain -C-C- single bond, and the peak at 962 cm^−1^ corresponds to -CH_2_- rocking vibration. These peaks indicate that the main component of the cable insulation is the PVC polymer. Additional additives can also be observed. The peak at 1420 cm^−1^ is assigned to the CO_3_ group of calcium carbonate, an important filler in PVC cable insulation. The absorption peak at 1735 cm^−1^ corresponds to carbonyl or ester groups, and the peak at 1580 cm^−1^ corresponds to aromatic -C-C- bonds. These peaks are associated with phthalate or terephthalate plasticizers, indicating their presence on the insulation surface.

After long-term overheating, most strong absorption peaks in the infrared spectrum of cable PVC insulation weakened to varying degrees. For example, the peak at 1420 cm^−1^ assigned to the -CH_2_-CHCl- structure and the three C-Cl stretching peaks in the 600–700 cm^−1^ region all weakened. The attenuation of chlorine-related absorption peaks indicates that, after continuous exposure to high temperatures, PVC side groups on the insulation surface lose chlorine atoms and form polyene structures, transforming the PVC main chain into a more unsaturated hydrocarbon structure.

**Figure 13:**
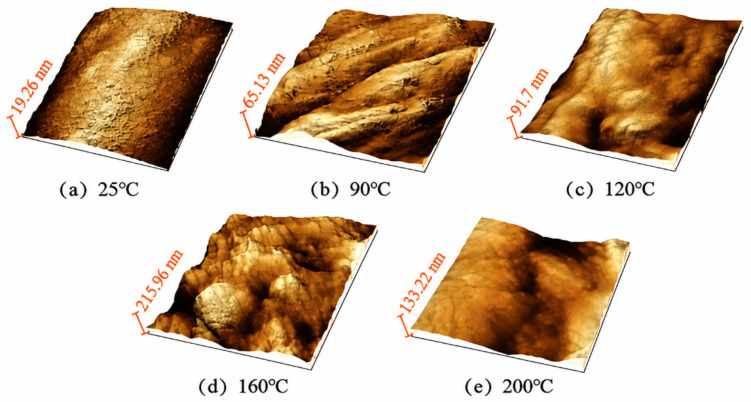
Surface morphology and potential distribution of PVC insulation after thermal degradation at different temperatures.

KPFM was used to characterize surface morphology and potential distribution after thermal degradation at different temperatures. As shown in Figure 13, the unaged outer-jacket surface was relatively uniform and flat, whereas increasingly pronounced protrusions and grooves developed after overheating. The average surface roughness increased from 7.698 to 13.555, 16.074, 27.337, and 43.390 nm as the treatment temperature increased. Thermal stress, oxidation, local deformation, and additive redistribution or loss are plausible contributors. Previous work has shown that temperature differences can generate internal mechanical stress, produce surface microcracks, and facilitate additive loss through crack channels [39].

### 4.2. Dielectric Deterioration

**Figure 14:**
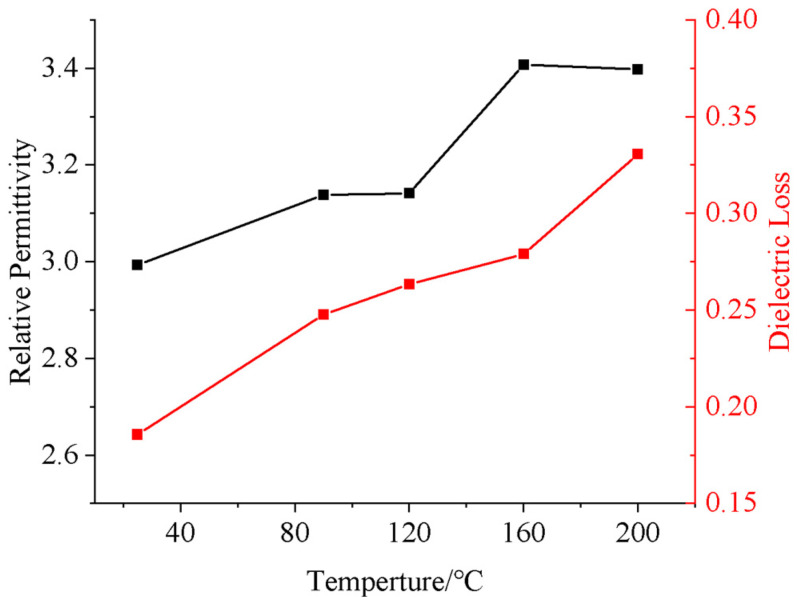
Variation trends of the relative dielectric constant and dielectric loss at power frequency under different degradation temperatures.

Dielectric properties changed systematically with degradation temperature. As shown in Figure 14, at a power frequency of 50 Hz, the real part of the dielectric constant increased by up to 13.9% relative to the room-temperature value. This increase is consistent with several concurrent effects: microcracks and pores can introduce additional interfaces and enhance interfacial polarization; thermal oxidation can change the population of polar groups such as carbonyl and hydroxyl groups; and elevated temperature can increase dipole mobility. These interpretations are physically plausible, but the dielectric measurement alone does not identify which chemical process dominates.

The dielectric loss factor at power frequency also increased with degradation temperature. This behavior is consistent with enhanced charge-carrier transport, additional interfacial polarization, and changes in the distribution of polar species. Plasticizer redistribution or loss is one plausible contributor [39]. The increase in loss factor leads to intensified energy dissipation and insulation-performance deterioration.

### 4.3. Coupled Degradation Pathway

**Figure 15:**
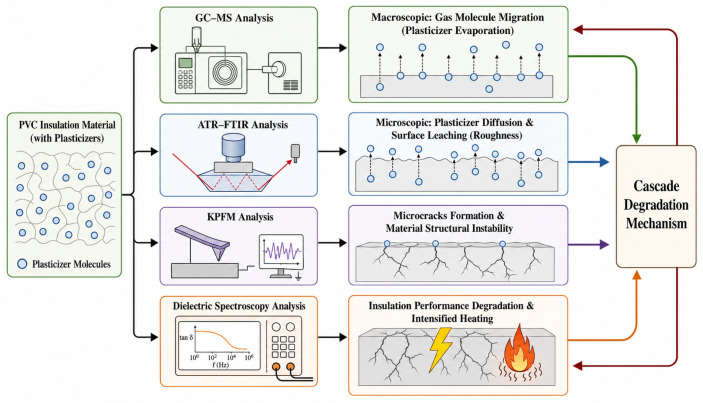
Schematic of the experimentally supported coupled pathway linking marker-gas evolution, structural instability, and dielectric-performance deterioration in the tested PVC cable outer jacket.

PVC degradation generally proceeds through dehydrochlorination, conjugated-polyene formation, and subsequent scission, crosslinking, cyclization, and aromatic-product formation [28,33,34,35]. Figure 15 summarizes how these established chemical processes are coupled with marker-gas evolution, surface change, and dielectric deterioration in the tested cable outer jacket.

The GC-MS results show that, over the 90–200 °C range, the observed organic profile is dominated by additive-related compounds at lower severity, followed by stronger contributions from PVC-chain transformation products at higher severity. Migration and volatilization of additive-related compounds are consistent with temperature-dependent diffusion, whereas the delayed benzene response is consistent with progression of dehydrochlorination and aromatization.

The ATR-FTIR changes are consistent with redistribution or loss of plasticizer-related species together with PVC-chain transformation; however, ATR-FTIR alone cannot determine the transport direction or distinguish migration from volatilization. Changes in ester/carbonyl and chlorine-related bands are therefore interpreted together with the GC-MS observations.

KPFM shows that overheating is accompanied by increasing surface roughness and non-uniform surface morphology. These measurements establish the development of surface defects. Plasticizer redistribution or loss, thermal stress, oxidation, and local deformation are plausible contributors and are interpreted jointly with the GC-MS and ATR-FTIR observations.

Dielectric testing establishes functional deterioration of the tested outer jacket as overheating severity increases. The increases in permittivity and dielectric loss are consistent with additional interfaces, changes in polar groups, and enhanced conduction or polarization loss. Their role in the coupled pathway is to relate observed gas and surface changes to insulation-performance deterioration under the same material and thermal conditions.

The coupled pathway is supported by mutually consistent observations. Early 2-EH release and later observation of DOTP/DEHA are consistent with additive-related migration and volatilization; delayed benzene formation and weakened chlorine-related ATR-FTIR bands are consistent with progression of PVC-chain transformation; and the increase in KPFM roughness and dielectric changes show that these chemical and transport processes co-evolve with surface damage and functional deterioration.

More specifically, the observations map onto three linked modules in Figure 15. In the gas-molecule migration module, 2-EH and the temperature-dependent DOTP/DEHA responses indicate additive redistribution and volatilization, while delayed benzene formation indicates an increasing contribution from PVC-chain transformation at higher severity. In the material-instability module, ATR-FTIR changes and KPFM roughness growth indicate that molecular changes, additive redistribution, and surface defects develop together. In the dielectric-deterioration module, increases in permittivity and loss indicate enhanced interfacial polarization and energy dissipation. Together, these modules link the observed gas, surface, structural, and electrical responses for the tested cable layer.

## 5. Discussion and Conclusions

Overheating-driven degradation of cable-grade PVC is both a scientific and practical concern because it accelerates dielectric deterioration and ultimately fire risk. Recent studies have examined gas-based cable-overheating diagnosis using different cable constructions, polymers, thermal protocols, and sensing methods [3,7,9,20,21,22]. Marker identity and response hierarchy vary with the cable construction, analysed layer, formulation, sampling protocol, ventilation, and sensing method.

In this work, a temperature–time–gas evidence chain was constructed for the PVC outer jacket of a YJV22 medium-voltage cable over 90–200 °C using headspace GC-MS response conversion. Gas evolution was compared with ATR-FTIR, KPFM, and dielectric deterioration. The results indicate a sequence governed by additive migration/volatilization and increasingly important PVC-chain transformations. The analysis provides a material-specific interpretation of the 2-EH/DOTP/DEHA/benzene marker system rather than a general marker set for all PVC cables.

The results extend qualitative identification by showing condition-dependent changes in estimated headspace levels within the tested temperature–time grid. 2-EH remained at or below approximately 50 × 10^−6^ up to 140 °C and reached approximately 300 × 10^−6^ at 200 °C for 60 min. Benzene was mainly observed at severe temperatures, and DOTP was first observed at 140 °C among the tested conditions, rising from approximately 40 × 10^−6^ at 140 °C for 5 min to more than 300 × 10^−6^ under more severe conditions.

For engineering interpretation, the present results may be organized into laboratory-derived indicative stages for this cable material: a baseline or trace-marker state at 90 °C or below within the tested protocol; an early indicative state from approximately 120 °C to below 140 °C, dominated by low-level 2-EH with little benzene; an intermediate warning-indicative state from approximately 140 °C to below 180 °C, characterized by the observation of DOTP/DEHA together with increasing 2-EH; and a severe indicative state at approximately 180–200 °C, associated with rapid increases in 2-EH, DOTP/DEHA, and benzene. These descriptive stages translate the compound-resolved laboratory trends into a marker sequence that can be evaluated in subsequent sensor studies. Chemical-sensor fire-detection research emphasizes early volatile signals, marker selectivity, multivariate discrimination, and control of nuisance backgrounds [5,7,20,21,22,40].

These gas trends align with independent evidence of deterioration. KPFM shows a monotonic roughness increase from 7.698 to 43.390 nm, and real permittivity increases by up to 13.9% at 50 Hz. Together, the results support a coupled pathway from additive-related emissions and PVC-chain transformation to surface damage and dielectric deterioration in the tested cable outer jacket.

Future work will evaluate the marker sequence using multi-point low-concentration calibration, current-loaded cables, in situ gas sampling, and independent sensor measurements under controlled ventilation and humidity.

In summary, this study provides temperature- and time-resolved headspace estimates for organic markers released from the PVC outer jacket of a YJV22-8.7/15 kV medium-voltage cable over 90–200 °C. The observed 2-EH, DOTP, DEHA, and benzene trends are accompanied by increasing surface roughness and dielectric deterioration. The resulting indicative stages summarize the marker sequence observed for this cable material and provide a basis for subsequent sensor evaluation.

## Figures and Tables

**Table 1:** Direct comparison of cable material/layer, thermal protocol, reported markers, analytical output, and threshold or validation status in gas-based cable-overheating studies.

Study/Material and Analyzed Layer	Thermal Protocol	Analytical/Diagnostic Output	Principal Markers	Threshold or Validation Status
Present study: YJV22-8.7/15 kV-3 × 185; PVC outer jacket only	Isothermal 90–200 °C; 5–60 min	Headspace GC-MS with single-point external-standard conversion; ATR-FTIR, KPFM and dielectric tests	2-EH, DOTP, DEHA, 2-EBe, 2-EBu and benzene	Compound-specific LOD/LOQ not determined; laboratory headspace levels only; no field-alarm threshold
Sugitani et al. [9]: 600 V commercial cable; mixed PVC insulation/sheath fragments	GC-MS at room temperature, 50, 90 and 120 °C for 1 h; chamber sensing at 20–120 °C	MonoTrap-GC-MS peak areas; SnO2 sensing and logistic-regression classification	Acetophenone, 2-phenyl-2-propanol, 2-EH, hexanal, alpha-methylstyrene, pentanal, 2-hexanone and 3-heptanone	GC-MS LOD/LOQ and field threshold not reported; classification accuracy 100% at 50 °C and 95% at 90/120 °C
Liu et al. [20]: heat-resistant PVC low-voltage cable and two XLPE medium-voltage cables	GC-MS screening at 270 °C for 15 min; sensor tests from room temperature to 260 °C	GC-MS compositional screening; 16-channel odor array and machine learning	Broad cable-specific odor fingerprints rather than an absolute marker concentration-time map	130 °C used as an abnormal boundary; about 90% recall and 70% precision; not a compound-specific analytical threshold
Zhang et al. [3]: actual inner and outer PVC cable layers	Programmed TG-FTIR from about 50 to 800 °C in air/Ar; electrical-stress experiments	TG-FTIR, GC/GC-MS, molecular simulation and gas-ratio analysis	HCl, CO, CO_2_, C_2_H_2_, alkanes and plasticizer fragments	Main decomposition about 200–350 °C depending on layer/atmosphere; no early-stage field-alarm threshold
Meng et al. [21]: ZC-BV10 PVC sheath	Discrete 5 min GC-MS conditions within 90–200 °C; sensor tests at 70–200 °C	GC-MS area normalization; 16-sensor array and Extra Trees classification	DEHP, DBP and 4-tert-octylphenol	Normal 70–90 °C, warning 120–150 °C and alarm 170–200 °C; 97% validation accuracy; boundaries are not GC-MS LOD/LOQ
Cao et al. [22]: high-voltage-cable PE outer sheath (different polymer)	90–250 °C at 10 °C increments; 180 min exposure	FTIR/micro-GC external-standard quantification and Random Forest classification	CO, CO_2_ and C2–C3 light hydrocarbons	Attention 90–130 °C, alert 140–180 °C and alarm 190–250 °C; 93.75% test accuracy

**Table 2:** Typical formulation ranges for cable-grade PVC.

Component	Content (Based on the Weight of PVC = 100)
PVC	100
Plasticizer	30–60
Stabilizer	5–15
Filler	5–15
Lubricant	1
Antioxidant	1
Colorant	<1

## Data Availability

The data presented in this study are available from the corresponding author upon reasonable request.

## Abbreviations

The following abbreviations are used in this manuscript:

ATR-FTIR	Attenuated total reflection Fourier-transform infrared spectroscopy
DEHA	Bis(2-ethylhexyl) hexanedioate
DOTP	Di(2-ethylhexyl) terephthalate
GC-MS	Gas chromatography-mass spectrometry
KPFM	Kelvin probe force microscopy
PVC	Poly(vinyl chloride)
TGA	Thermogravimetric analysis
2-EH	2-Ethylhexanol
